# Supplementary material for: Associated factors with adherence to preventive behaviors related to COVID-19 among medical students in the university of Monastir, Tunisia
Source: PLoS One. 2023 Mar 14;18(3):e0280921. doi: 10.1371/journal.pone.0280921 (PMC10013900; doi:10.1371/journal.pone.0280921)
Supplement: S1 File — (PDF) [file pone.0280921.s001.pdf]

L'Échelle du stress perçu

Pour chaque question, choisissez parmi les alternatives suivantes :

0 - jamais 1 - presque jamais 2 - parfois 3 - assez souvent 4 - souvent

	Jamais	Presque jamais	Parfois	Assez souvent	Souvent
1. Au cours du dernier mois combien de fois, avez-vous été dérangé (e) par un évènement inattendu ?	0	1	2	3	4
2. Au cours du dernier mois combien de fois vous a t-il semblé difficile de contrôler les choses importantes de votre vie ?	0	1	2	3	4
3. Au cours du dernier mois combien de fois vous êtes-vous senti(e) nerveux (se) ou stressé(e) ?	0	1	2	3	4
4. Au cours du dernier mois combien de fois vous êtes-vous senti(e) confiant(e) à prendre en main vos problèmes personnels ?	0	1	2	3	4
5. Au cours du dernier mois combien de fois avez-vous senti que les choses allaient comme vous le vouliez ?	0	1	2	3	4
6. Au cours du dernier mois combien de fois avez-vous pensé que vous ne pouviez pas assumer toutes les choses que vous deviez faire ?	0	1	2	3	4
7. Au cours du dernier mois combien de fois avez-vous été capable de maîtriser votre énervement ?	0	1	2	3	4
8. Au cours du dernier mois combien de fois avez-vous senti que vous dominiez la situation ?	0	1	2	3	4
9. Au cours du dernier mois combien de fois vous êtes-vous senti(e) irrité(e) parce que évènements échappaient à vôtre contrôle ?	0	1	2	3	4
10. Au cours du dernier mois combien de fois avez-vous trouvé que les difficultés s'accumulaient à un tel point que vous ne pouviez les contrôler ?	0	1	2	3	4

Calcul du score PSS :

• Commencez par inverser les scores pour les questions 4, 5, 7 et 8. Sur ces 4 questions, modifiez les scores comme ceci : 0 = 4, 1 = 3, 2 = 2, 3 = 1, 4 = 0.

• Maintenant, additionnez les scores pour chaque élément pour obtenir le score total.

Le score total peut varier de 0 à 40.

• Les scores allant de 0 à 13 seraient considérés comme un stress perçu faible.

• Les scores allant de 14 à 26 seraient considérés comme un stress perçu modéré.

• Les scores allant de 27 à 40 seraient considérés comme un stress perçu élevé.
