# Supplementary material for: Associated factors with adherence to preventive behaviors related to COVID-19 among medical students in the university of Monastir, Tunisia
Source: PLoS One. 2023 Mar 14;18(3):e0280921. doi: 10.1371/journal.pone.0280921 (PMC10013900; doi:10.1371/journal.pone.0280921)
Supplement: S2 File — (PDF) [file pone.0280921.s002.pdf]

Le Questionnaire sur le trouble anxieux en sept points (GAD-7)

GAD-7

Au cours des 2 dernières semaines, à quelle fréquence avez-vous été dérangé(e) par les problèmes suivants?	*Jamais*	*Plusieurs jours*	*Plus de la moitié du temps*	*Presque tous les jours*
1. Sentiment de nervosité, d'anxiété ou de tension	0	1	2	3
2. Incapable d'arrêter de vous inquiéter ou de contrôler vos inquiétudes	0	1	2	3
3. Inquiétudes excessives à propos de tout et de rien	0	1	2	3
4. Difficulté à se détendre	0	1	2	3
5. Agitation telle qu'il est difficile de rester tranquille	0	1	2	3
6. Devenir facilement contrarié(e) ou irritable	0	1	2	3
7. Avoir peur que quelque chose d'épouvantable puisse arriver	0	1	2	3

Score total _______ = Ajouter les colonnes _______ + _______ + _______

Si vous avez coché au moins un des problèmes, dans quelle mesure ce ou ces problèmes ont-ils rendu difficiles votre travail, vos tâches à la maison ou votre capacité à bien vous entendre avec les autres?

❑ Pas du tout difficile ❑ Plutôt difficile ❑ Très difficile ❑ Extrêmement difficile

Niveau de gravité basé sur le score au GAD-7

SCORE AU GAD-7	NIVEAU DE GRAVITÉ DU TROUBLE ANXIEUX
De 0 à 4	Minimal
De 5 à 9	Léger
De 10 à 14	Modéré
De 15 à 21	Grave

D'après Spitzer RL, Kroenke K, Williams JBW, Löwe B. A Brief Measure for Assessing Generalized Anxiety Disorder: The GAD-7. *Arch Intern Med*. 2006;166(10):1092–1097. doi:10.1001/archinte.166.10.1092
