# Supplementary material for: Associated factors with adherence to preventive behaviors related to COVID-19 among medical students in the university of Monastir, Tunisia
Source: PLoS One. 2023 Mar 14;18(3):e0280921. doi: 10.1371/journal.pone.0280921 (PMC10013900; doi:10.1371/journal.pone.0280921)
Supplement: Attachment — Submitted filename: Response to reviewers.docx [file pone.0280921.s004.docx]

**Response to Reviewers**

Dear Editor,

Thank you for your attention to our work. The comments and suggestions we have received are valuable and very helpful to improve our manuscript. We have made revisions based on your latest comments and suggestions, as described in the authors' response.

We respond in detail to the reviewers’ comments. They raised important issues and we agree with almost all their comments. We have revised our manuscript according to their indications. We hope that they will find our responses to their comments satisfactory, and we are willing to finish the revised version of the manuscript including any further suggestion that the reviewers may have. The revision has been developed in consultation with all coauthors, and each author has given approval to the final form of this revision. All changes made in the revised version will be visible in red.

Please find as attached files:

The “Response to Reviewers”,

The “Revised Manuscript with Track Change” and

the “Manuscript” (the unmarked version of the revised manuscript).

We sincerely hope that the revision of the manuscript will be satisfactory, and the enclosed version will be acceptable for publication.

Once again thank you for your cooperation.

Sincerely Yours,

Dr Imen ZEMNI

**Journal Requirements:**

1. Please ensure that your manuscript meets PLOS ONE's style requirements, including those for file naming.

**Response:** Thank you, we have done the required modifications.

2. In your Data Availability statement, you have not specified where the minimal data set

underlying the results described in your manuscript can be found. PLOS defines a study's minimal data set as the underlying data used to reach the conclusions drawn in the manuscript and any additional data required to replicate the reported study findings in their entirety. All PLOS journals require that the minimal data set be made fully available.

**Response:** Thank you, we have uploaded data as a supporting information file (S3. File).

3. Please review your reference list to ensure that it is complete and correct. If you have cited

papers that have been retracted, please include the rationale for doing so in the manuscript text or remove these references and replace them with relevant current references. Any changes to the reference list should be mentioned in the rebuttal letter that accompanies your revised manuscript. If you need to cite a retracted article, indicate the article’s retracted status in the References list and also include a citation and full reference for the retraction notice.

**Response:** Thank you, we have made the necessary revisions.

**Review Comments:**

**Reviewer #1:**

Dear authors, very good work. Only some simple points:

Methods:

1. The phrase” Were included in the study, all undergraduate medical students of the faculty of medicine of Monastir from the first year to the fifth year.” on page 13, line 4, needs to be rearranged linguistically. In my opinion, it can be changed into “All undergraduate medical students of the faculty of medicine of Monastir from the first year to the fifth year were included in the study.”

**Response:** Thank you, we have done the required modifications. (Lines 115-116)

2. I suggest to mention the scores of the GAD 7 scale and PSS-10 in methods.

**Response:** Thank you, we have added supporting files detailing the GAD 7 scale and the PSS-10 scale (S1. File and S2. File) and we have better detailed the interpretation of the scores. (Lines 128 -131)

4. Are the scores of the adherence only good (80-100%) or bad (less than 80%), not moderate? **Response:** Thank you, we have categorized compliance with precautionary measures related to COVID-19 using Bloom’s cut-off point, as “good” if the score was between 80 and 100%, moderate if the score was between 60 and 79%, and poor if the score was less than 60%. We have added these details in the methods section (Lines 143-145) and we have represented the results of these three classes in Figure 2.

Results:

In table 1:

a. The sum of percentages of marital status is 99.77, not 100%, you must modify?

b. The sum of percentages of stress is 99.9, not 100%, you must modify?

**Response:** Thank you, we have made the necessary corrections (Table 1).

**Reviewer #2:**

I appreciate your efforts in this study. Generally, speaking, the work is technically acceptable, and the defects could be amended in the review process. However, considering the rapidly evolving the public knowledge and practice towards COVID-19, I'm afraid publishing a survey which is conducted 2 years ago would be futile. It's very likely that the same respondents' current practice and knowledge is very different from 2 years ago. This study would neither reflect the current situation nor assist policy makers in making sound, updated decisions. I wish the authors could publish the work shortly after the study was performed. A delay in publishing surveys on many chronic conditions can be ignored, as the public perceptions and practice may vary slowly over time. But KAP studies about an infectious pandemic, especially when the causative agent, and subsequently the disease manifestations and our knowledge about that, are highly variating, should be published as soon as possible. Otherwise, the benefits of disseminating the findings of the study would not be achieved.

**Response:** Thank you, we agree with you.

We have tried to explain the impact and usefulness of this study for the future (lines 388 - 394):

We think that the results of this study would be very useful for the future since we have been seeing today more and more epidemics of communicable diseases (coronavirus, influenzae, monkeypox...).

Our study has identified categories with poor adherence to precautionary measures among medical students. Awareness programs should target especially these students in future epidemic contexts.
